# Blockade of dengue virus infection and viral cytotoxicity in neuronal cells *in vitro* and *in vivo* by targeting endocytic pathways

**DOI:** 10.1038/s41598-017-07023-z

**Published:** 2017-07-31

**Authors:** Min-Ru Ho, Tsung-Ting Tsai, Chia-Ling Chen, Ming-Kai Jhan, Cheng-Chieh Tsai, Yi-Chao Lee, Chun-Han Chen, Chiou-Feng Lin

**Affiliations:** 1 Department of Microbiology and Immunology, School of Medicine, College of Medicine, Taipei Medical University, Taipei, 110 Taiwan; 2 Graduate Institute of Medical Sciences, College of Medicine, Taipei Medical University, Taipei, 110 Taiwan; 3 Translational Research Center, Taipei Medical University, Taipei, 110 Taiwan; 4 Department of Nursing, Chung Hwa University of Medical Technology, Tainan, 717 Taiwan; 5 The PhD Program for Neural Regenerative Medicine, College of Medical Science and Technology, Taipei Medical University, Taipei, 110 Taiwan; 6 Department of Pharmacology, School of Medicine, College of Medicine, Taipei Medical University, Taipei, 110 Taiwan

## Abstract

Dengue virus (DENV) infection in neuronal cells was speculated to trigger neuropathy. Herein, we determined the blockade of DENV infection by targeting endocytic pathways *in vitro* and *in vivo*. In DENV-infected mouse brains, we previously showed that viral proteins are expressed in neuronal cells around the hippocampus with accompanying neurotoxicity. DENV caused infection, including entry, double-stranded (ds)RNA replication, protein expression, and virus release, followed by cytotoxicity in the mouse neuronal Neuro-2a cell line. Pharmacologically blocking clathrin-mediated endocytosis of the DENV retarded viral replication. Targeting vacuolar-type H^+^-ATPase (V-ATPase)-based endosomal acidification effectively blocked the DENV replication process, but had no direct effect on viral translation. Blockade of the clathrin- and V-ATPase-based endocytic pathways also attenuated DENV-induced neurotoxicity. Inhibiting endosomal acidification effectively retarded DENV infection, acute viral encephalitis, and mortality. These results demonstrate that clathrin mediated endocytosis of DENV followed by endosomal acidification-dependent viral replication in neuronal cells, which can lead to neurotoxicity.

## Introduction

Dengue virus (DENV), a positive-sense single-stranded RNA virus of the family Flaviviridae, causes approximately 50 million infections per year^1^. The DENV genome encodes structural proteins (capsid (C), pre-membrane (prM), and envelope (E) proteins) and nonstructural proteins (NS1, NS2A, NS2B, NS3, NS4A, NS4B, and NS5) that are involved in viral infection and pathogenesis^2^. Through putative receptors, such as heparan sulfate, mannose receptor, dendritic cell-specific ICAM-grabbing non-integrin (CD209), heat shock protein 90/70, phosphatidylserine receptors, etc., DENV causes various infections that are both cell-specific and receptor-dependent^3^. Different serotypes of DENV cause clathrin-dependent and -independent endocytosis, and various cell types of the DENV infection entry pathway occur through a non-classical endocytic pathway, independent of caveolae and lipid rafts^4^. Following viral receptor-mediated endocytosis accompanied by vacuolar-type hydrogen (H^+^)-ATPase (V-ATPase)-mediated endosomal acidification, viral RNA can be uncoated from the enveloped virus and released into the cytoplasm for protein translation and genome replication^3^. Targeting the endocytic pathways of DENV infection, including viral entry/fusion and uncoating, confers a wide range of antiviral effects^3, 5, 6^.

Flaviviral encephalitis, including Japanese encephalitis, West Nile encephalitis, St. Louis encephalitis, Murray Valley encephalitis, etc., is well recognized in endemic regions, clinically characterized by a reduced level of consciousness associated with seizures, poliomyelitis-like paralysis, and parkinsonian movement disorders^7^. The emergence of neurotropic arboviruses, such as Zika virus, chikungunya virus, and DENV, is associated with acute neurological complaints^8, 9^. In severe dengue infection, symptoms include plasma leakage, mucosal bleeding, neurological dysfunction, and multiple organ failure^1, 2^. Although DENV is not a characteristic neurotropic virus, dengue is widely considered to be one of the leading causes of neurological manifestations, including encephalitis, encephalopathy, dengue-associated neuromuscular complications, and dengue-associated neuro-ophthalmic complications^10–12^. However, the pathogenesis of dengue-associated neurological complications is not fully understood due to a lack of appropriate evaluations in previous studies. A retrospective 2-year study reported that dengue patients may show various persistent neurological complications^13^. DENV is rapidly replicated and causes viremia during the acute phase of infection, and it was speculated that acute viral encephalitis and central nervous system (CNS) inflammation may facilitate dengue-associated neurological complications.

Intracranial infection with non-adapted DENV induces lethality in immunocompetent mice following limb paralysis, seizures, and encephalitis^14–16^. Expressions of viral antigens, double-stranded (ds)RNA, and virus particles are found in DENV-infected brains. Importantly, *in vivo* DENV infection in mouse brains causes neuronal damage in the pyramidal layer of Cornu Ammonis (CA) areas of the hippocampus^14, 16^. Based on viral antigen identification, neurons can be infected by DENV; however, viral receptors and infectious routes have not been well addressed. The targeting of dopamine receptors, which are expressed in hippocampal neurons^17^, retarded DENV infection *in vitro* ^18, 19^ and *in vivo* ^19^. Studies showed induction of neuronal cell death, particularly apoptosis, by DENV infection both *in vitro* ^20–23^ and *in vivo* ^14, 16, 24^. This study attempted to investigate the infectious route of DENV in neuronal cells and evaluate potential antiviral strategies by targeting endocytic pathways of DENV infection *in vitro* and *in vivo*.

## Results

### DENV initiates infection in neuronal cells *in vitro*, including viral entry, RNA replication, protein expression, and viral release

**Figure 1:**
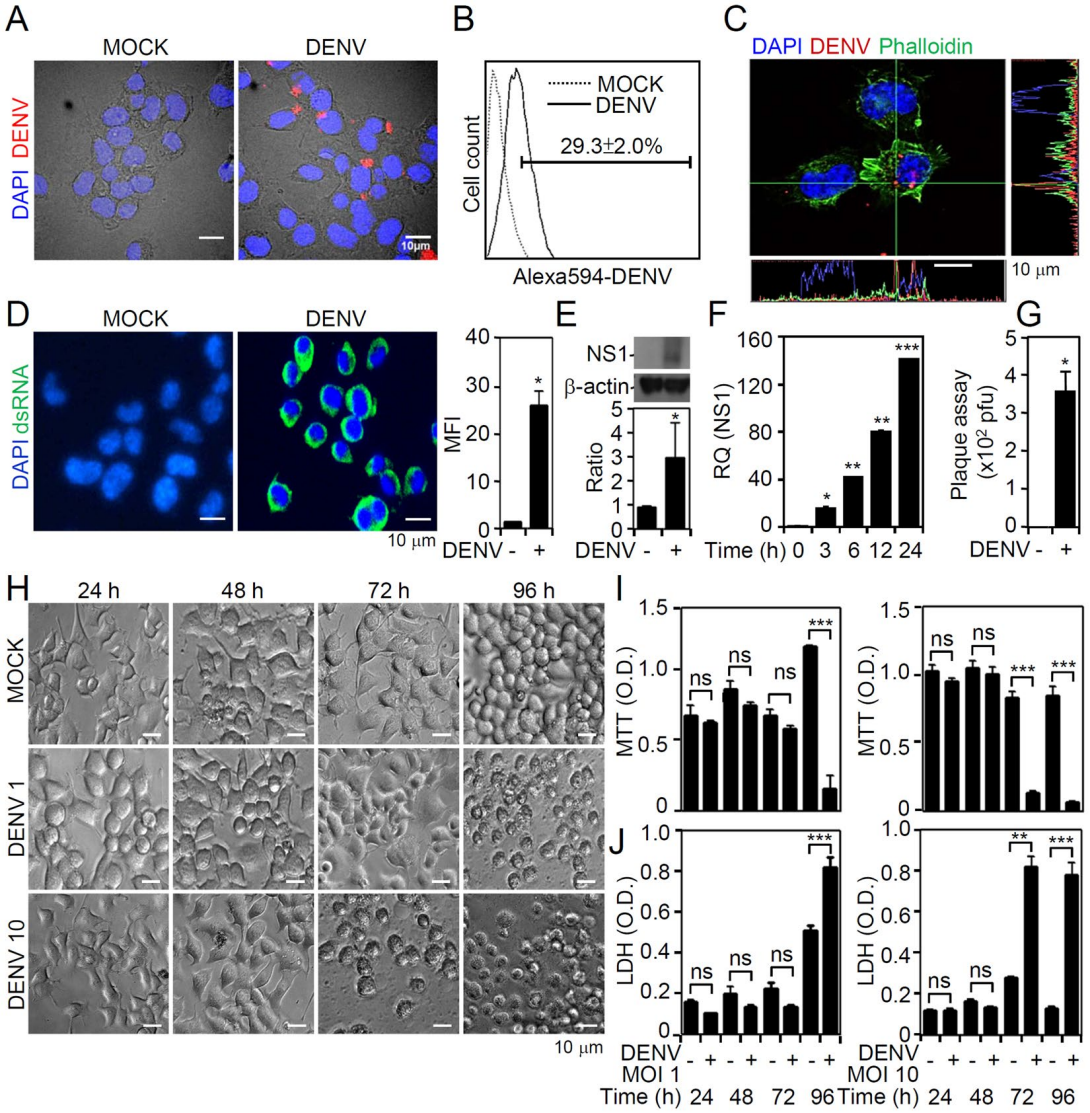
Dengue virus (DENV) serotype 2 PL046 (DENV2) causes infection, including viral entry, double-stranded (ds)RNA replication, viral protein expression, virus release, and neurotoxicity, in the Neuro-2a murine neuronal cell line. (**A**) Fluorescence, (**B**) flow cytometric, and (**C**) three-dimensional confocal image analyses showed Neuro-2a cells carrying Alexa-594 labeled (*red*) DENV2 at 2 h post-infection. Phalloidin (*green*) staining indicates actin. (**D**) Immunocytochemistry and the relative mean fluorescence intensity (*MFI*) of viral dsRNA (*green*) at 24 h post-infection. (**E**) The Western blot analysis showed viral nonstructural protein1 (*NS1*) expression at 24 h post-infection. The relative ratio to β-actin is shown. (**F**) A qPCR showed the time-kinetic mRNA expression of NS1, and (**G**) plaque assays showed the level of viral replication at 24 h post-infection. (**H**) Cell morphology, (**I**) MTT, and (**J**) lactate dehydrogenase (LDH) assays showed cell growth, viability, and cytotoxicity, respectively, in DENV-infected cells for the indicated times and different multiplicities of infection (MOIs). For all images and blots, representative data were selectively obtained from three individual experiments. DAPI staining indicates nuclei (*blue*). For the flow cytometric analysis, the percentage of positive cells is shown. All quantitative data are shown as the mean ± SD of three independent experiments. *p < 0.05, **p < 0.01, and ***p < 0.001. ns, not significant.

In DENV-infected brains of 7-day ICR mice^16^, we noted that NS3-positive cells were present within the Iba-1-negative cell population, suggesting the ability of DENV to infect non-microglia *in vivo*. Previous work^15, 24^ showed that DENV can infect neuronal cells *in vivo*; however, the effects of DENV on neuronal cells remain poorly understood. To demonstrate infection efficacy in Neuro-2a cells, we performed fluorescent DENV staining followed by fluorescent imaging (Fig. 1A) and a flow cytometric analysis (Fig. 1B). Results showed viral binding/entry at 2 h post-inoculation. To confirm this finding, we used confocal microscopy to evaluate the intracellular localization of fluorescence-stained DENV in Neuro-2a cells (Fig. 1C). To investigate DENV replication in neuronal cells, we used immunostaining, which demonstrated significant viral dsRNA expression in DENV-infected Neuro-2a cells (*p* < 0.05, Fig. 1D). We also performed Western blotting (Fig. 1E) and a quantitative polymerase chain reaction (qPCR) (Fig. 1F). Our results confirmed viral NS1 protein expression in Neuro-2a cells 24 h post-infection. A plaque assay, which was performed to determine viral replication and release, showed significant (*p* < 0.05) infection of Neuro-2a cells by DENV (Fig. 1G).

Following infection, DENV can cause neurotoxicity as shown by histopathological changes and apoptotic staining^16^. Changes in cell morphology, cell growth, and cytotoxicity were used to evaluate the effects of DENV on neuronal cells. Microscopic observations revealed a significant change in Neuro-2a cells after DENV infection (Fig. 1H). The MTT (Fig. 1I) and lactate dehydrogenase (LDH; Fig. 1J) assays showed that DENV caused cell growth inhibition and cytotoxicity, particularly when cells were incubated with a high multiplicity of infection (MOI). These results indicated that DENV can infect neuronal cells and cause neurotoxicity *in vitro*.

### D2R mediates DENV2 infection in Neuro-2a cells

Currently, the targeting of dopamine receptors retards DENV infection *in vitro* ^18, 19^ and *in vivo* ^19^, suggesting a potential role of dopamine receptor-mediated DENV infection. We performed immunostaining that revealed expression of dopamine receptor D2 (D2R), but not D4R, in Neruo-2a cells (Supplemental Fig. 1A). Additionally, immunostaining showed the expression of D2R in isolated NeuN-positive hippocampal neurons, a neuronal nuclear antigen that is commonly used as a biomarker for neurons (Supplemental Fig. 1B). Fluorescent DENV staining followed by fluorescent imaging showed significant viral binding/entry in isolated hippocampal neurons (Supplemental Fig. 1C). Plaque assays confirmed that significant DENV replication occurred in isolated hippocampal neurons (Supplemental Fig. 1D).

To verify the essential role of D2R in mediating DENV infection, we used metoclopramide (MCP) to pharmacologically decrease DENV binding/entry (Supplemental Fig. 1E) and viral replication (Supplemental Fig. 1F) in Neuro-2a cells, as respectively shown by fluorescent imaging and plaque assays. These findings indicated that DENV caused infection of neuronal cells in a D2R-mediated manner.

### DENV2 infects Neuro-2a cells via clathrin-mediated endocytosis

**Figure 2:**
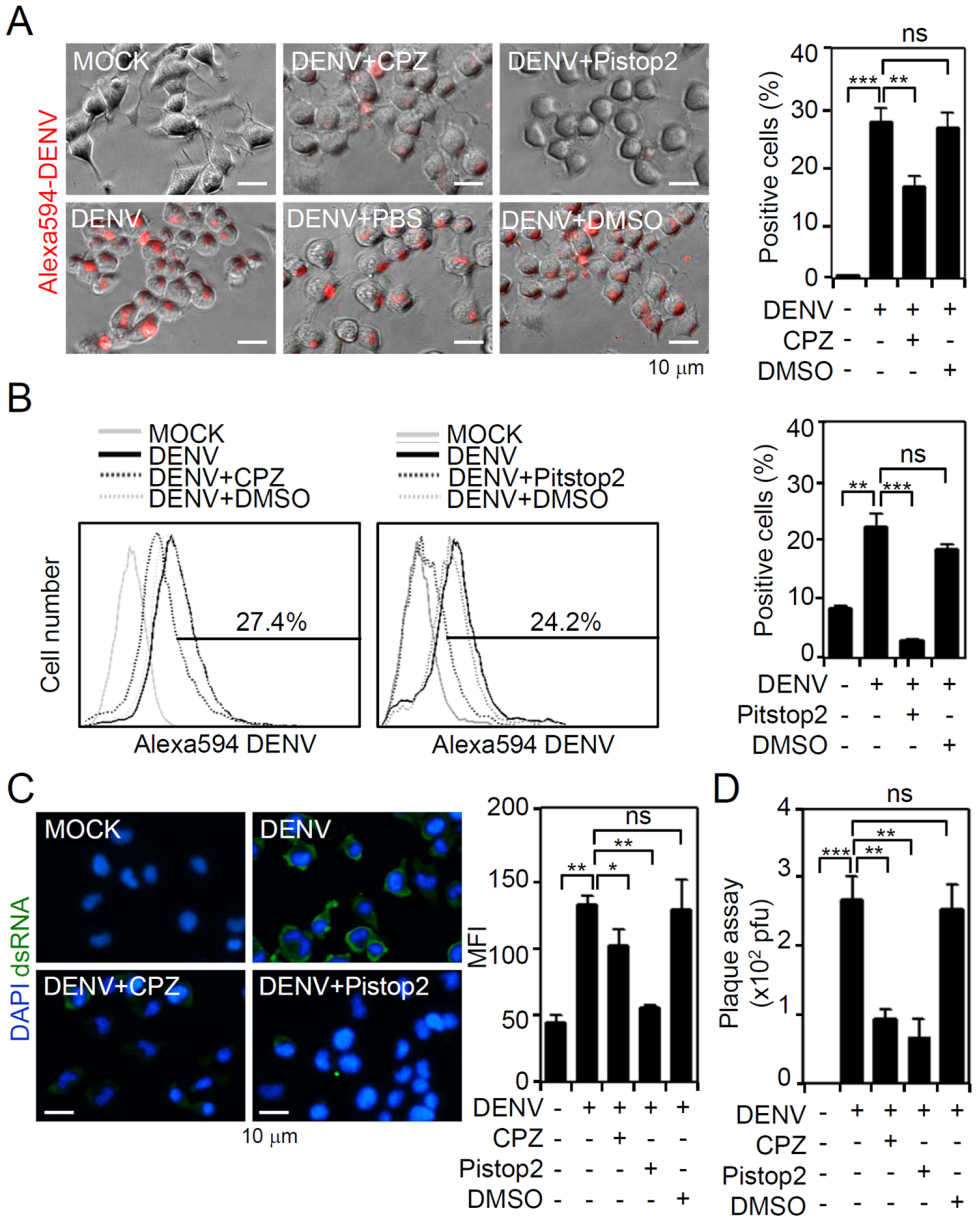
Inhibition of clathrin-mediated endocytosis partly reduces dengue virus serotype 2 (DENV2) infection in Neuro-2a cells. Neuro-2a cells were inoculated with Alexa-594-labeled DENV2 (at a multiplicity of infection of 1) for 2 h in the presence of the clathrin inhibitors, chlorpromazine (CPZ; 5 ng/ml) and Pitstop2 (30 µM). (**A**) Fluorescent microscopy and (**B**) flow cytometry were respectively used to measure the expression and percentage of cells carrying fluorescent DENV2. (**C**) Immunocytochemistry and the relative mean fluorescence intensity (*MFI*) of viral dsRNA (*green*) 24 h post-infection. (**D**) Plaque assays show the level of viral replication 24 h post-infection. For all images, representative data were selectively obtained from three individual experiments. Quantitative data are depicted as the mean ± SD. *p < 0.05, **p < 0.01, and ***p < 0.001. ns, not significant.

DENV infects cells via distinct entry pathways for DENV internalization, including clathrin-mediated and clathrin-independent endocytosis^3, 25, 26^. However, no further evidence has revealed the endocytic pathway of DENV in neuronal cells. To investigate the involvement of (clathrin-mediated) endocytosis, the pharmacological inhibitors, CPZ and Pitstop 2, were utilized as previously described^4^. We performed fluorescent DENV staining followed by fluorescent imaging (Fig. 2A) and a flow cytometric analysis (Fig. 2B). Treatment with CPZ and Pitstop 2 had significantly reduced viral binding/entry in Neuro-2a cells at 2 h post-inoculation. In confirmation of this finding, immunostaining demonstrated a significant decrease in viral dsRNA expression in DENV-infected Neuro-2a cells co-treated with CPZ and Pitstop 2 (Fig. 2C). We also performed plaque assays to study viral replication and release, and results showed significant (*p* < 0.01) inhibition in DENV-infected Neuro-2a cells (Fig. 2D). Treatment with CPZ and Pitstop 2 did not affect cell growth or cytotoxicity, but they caused an inhibition of acidification following DENV infection. These results show clathrin-regulated DENV binding/entry followed by viral replication in neuronal cells *in vitro*.

### Blockade of endosomal acidification attenuates DENV2 infection in Neuro-2a cells

**Figure 3:**
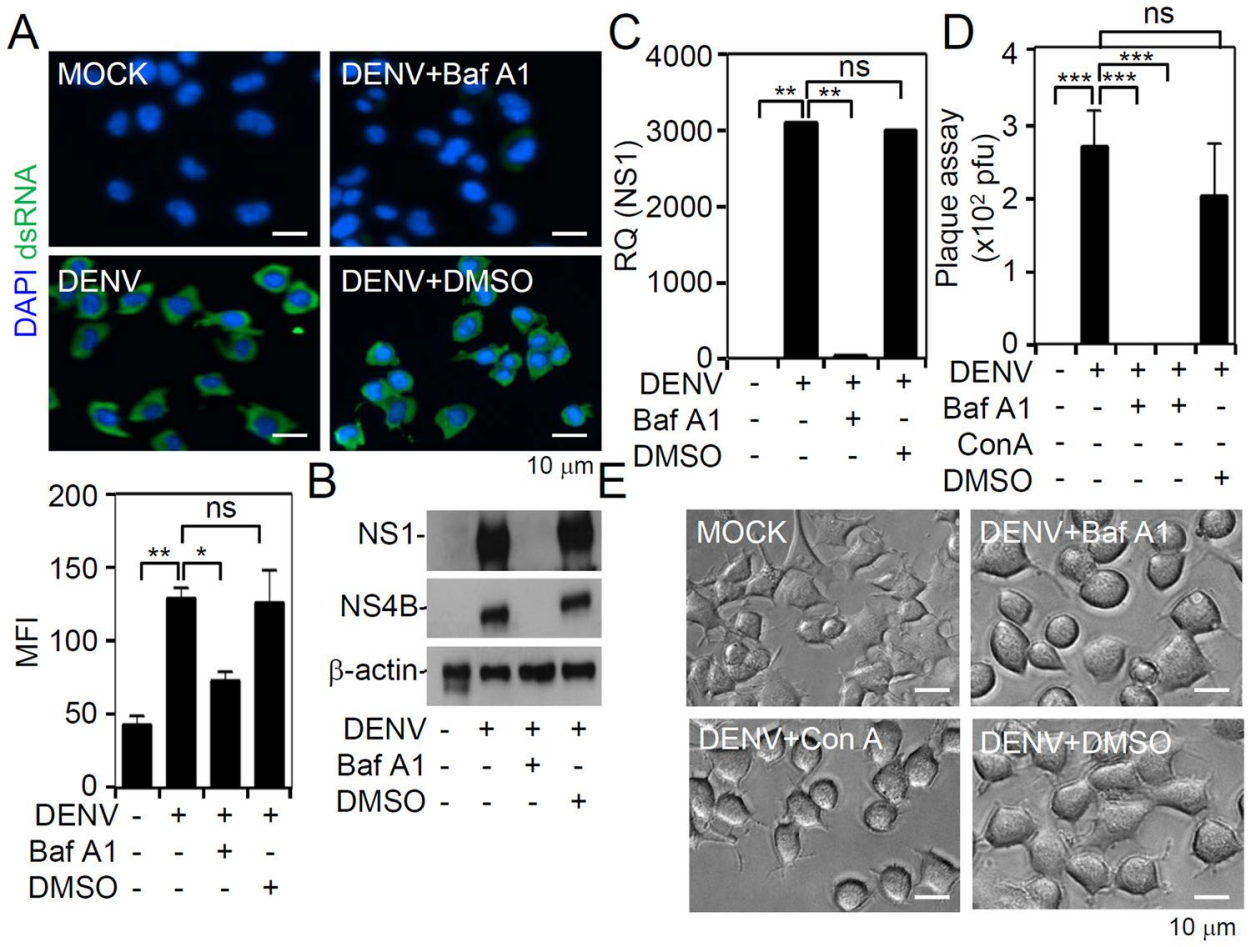
Inhibition of endosomal acidification effectively abolishes dengue virus serotype 2 (DENV2) infection *in vitro*. Neuro-2a cells were inoculated with DENV2 (at a multiplicity of infection of 1) for 24 h in the presence of the V-ATPase inhibitor, bafilomycin A1 (BafA1; 100 nM) or concanamycin A (ConA; 50 nM). (**A**) Immunocytochemistry and the relative mean fluorescence intensity (*MFI*) of viral dsRNA (*green*). (**B**) Western blotting analysis shows viral NS1 and NS4B expressions. The relative ratio to β-actin is shown. (**C**) qPCR shows the mRNA expression of NS1. (**D**) Plaque assays show the level of viral replication. (**E**) Cell morphology of DENV2-infected cells. For all images, representative data were selectively obtained from three individual experiments. Quantitative data are depicted as the mean ± SD of three independent experiments. *p < 0.05, **p < 0.01, and ***p < 0.001. ns, not significant.

Once the dengue viral receptor mediates endocytosis, V-ATPase facilitates endosomal acidification for an uncoating of the viral genome^3^. A previous study showed that the dengue prM protein interacts with V-ATPase to facilitate viral entry and egression^27^. We used BafA1 and ConA, which are inhibitors of V-ATPase^28^, to decrease endosomal acidification. Immunostaining demonstrated a blockade of viral dsRNA expression in DENV-infected Neuro-2a cells pretreated with BafA1 (*p* < 0.05, Fig. 3A). We also performed Western blotting, a qPCR, and plaque assays to respectively confirm that inhibiting endosomal acidification reduced viral NS1 protein expression (Fig. 3B), NS1 gene expression (Fig. 3C), and viral replication (Fig. 3D) in Neuro-2a cells at 24 h post-infection. No changes in cell morphology (Fig. 3E), cell growth, or cytotoxicity were observed after drug treatment followed by DENV infection at 24 h post-infection. Treatment with BafA1, ConA, and lysosomotropic agents, such as chloroquine and NH_4_Cl, reduced acidification. These results indicate that inhibiting endosomal acidification attenuated DENV infection in neuronal cells.

### Inhibition of endosomal acidification reduces DENV2 infection independent of translation

**Figure 4:**
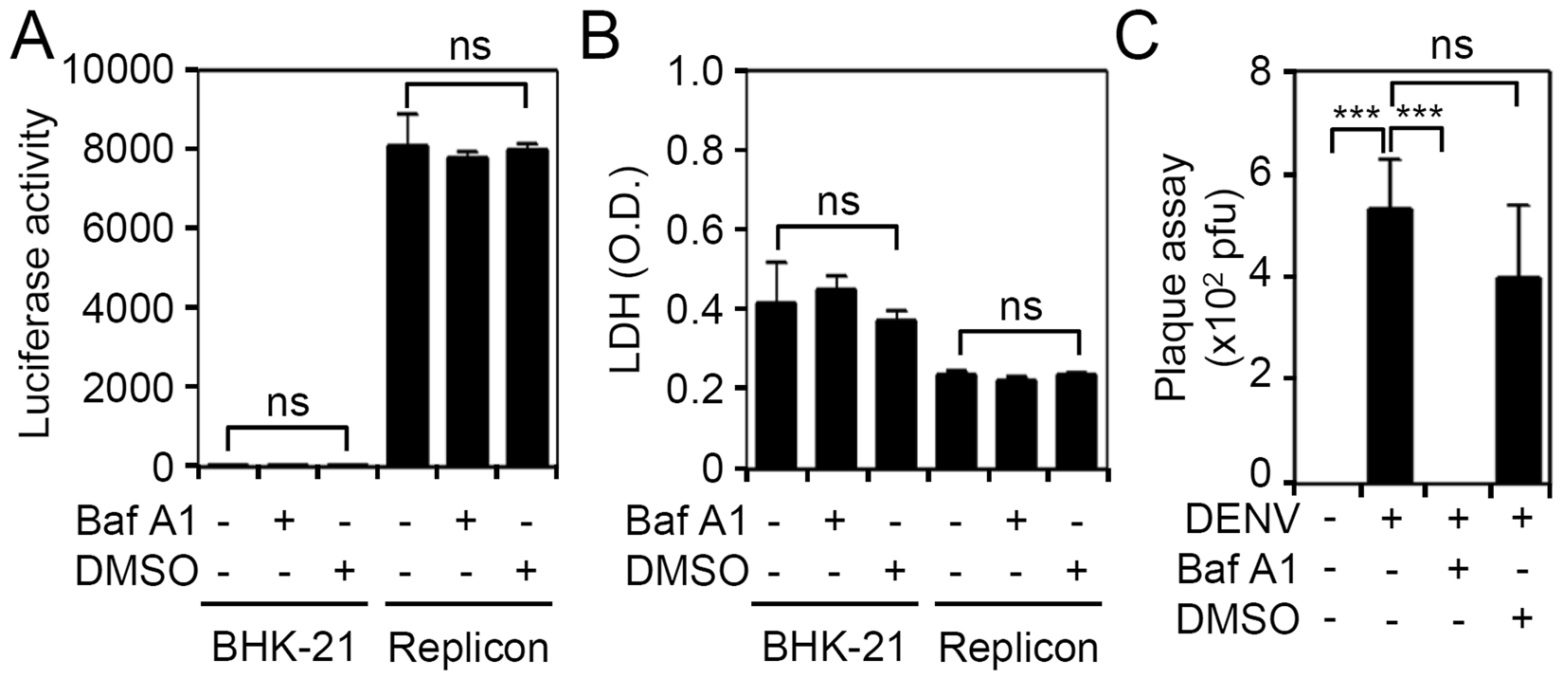
Inhibition of endosomal acidification does not repress firefly luciferase activity in BHK-D2-Fluc-SGR-Neo-1 cells. (**A**) Luciferase activity and (**B**) lactate dehydrogenase (LDH) assays in bafilomycin A1 (BafA1; 100 nM)-treated parental BHK-21 and BHK-D2-Fluc-SGR-Neo-1 cells (replicons) 24 h post-treatment. (**C**) Plaque assays show the level of viral replication 24 h post-infection in dengue virus serotype 2 (DENV2) (at a multiplicity of infection of 1)-infected BHK-21 cells with or without BafA1 (100 nM) at 0.5 h of pretreatment. Quantitative data are depicted as the mean ± SD of three independent experiments. ***p < 0.001. ns, not significant.

We confirmed the inhibitory role of endosomal acidification only in viral genome uncoating but not in other steps of the viral cell cycle, by using firefly luciferase activity in BHK-D2-Fluc-SGR-Neo-1 cells, and found that treatment with BafA1 caused no direct inhibitory effects on viral translation or replication (Fig. 4A) or cytotoxicity in cells (Fig. 4B). However, consistent with results in Neuro-2a cells, BafA1 caused blockade of DENV2 replication in parental BHK-21 cells, as shown by plaque assays (Fig. 4C). These results indicate that the blockade of endosomal acidification had no direct effects on viral inhibition via alterations of viral translation and replication.

### Targeting endocytic pathways reduces *in vitro* neurotoxicity induced by DENV infection

**Figure 5:**
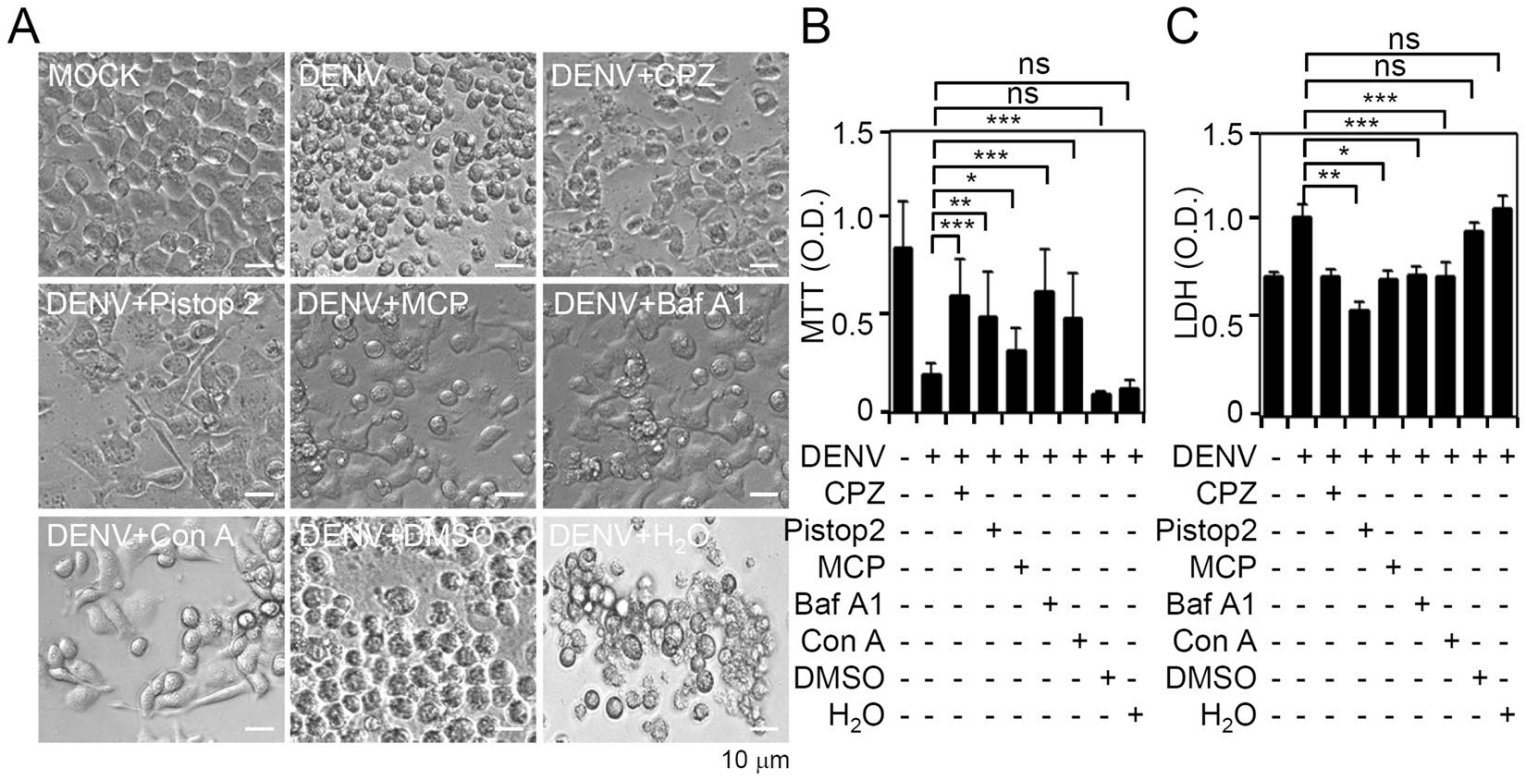
Pharmacologically targeting endocytic pathways reduces dengue virus serotype 2 (DENV2)-induced neurotoxicity *in vitro*. Following pretreatment with chlorpromazine (CPZ; 5 ng/ml), Pitstop2 (30 µM), metoclopramide (MCP; 10 µM), BafA1 (100 nM), or concanamycin A (ConA; 50 nM), (**A**) cell morphology, (**B**) MTT, and (**C**) lactate dehydrogenase (LDH) assays respectively show cell growth, viability, and cytotoxicity in DENV (at a multiplicity of infection of 10)-infected cells at 72 h. For all images, representative data were selectively obtained from three individual experiments. All quantitative data are shown as the mean ± SD of three independent experiments. *p < 0.05, **p < 0.01, and ***p < 0.001. ns, not significant.

Infection with DENV causes neuronal cell apoptosis^20–23^. To analyze roles of endocytic pathways involved in DENV infection in neuronal cells, we showed that pretreatment with CPZ, Pitstop2 (for blocking clathrin-mediated endocytosis), BafA1, and ConA (for inhibiting acidification) effectively abolished DENV-induced changes in cell morphology (Fig. 5A), cell growth inhibition (Fig. 5B), and cytotoxicity (Fig. 5C). Treatment with MCP (for inhibiting D2R) was used as a positive control, as D2R mediates DENV binding/entry. These results indicate the involvement of endocytic pathways in DENV-induced neurotoxicity *in vitro*.

### Inhibiting endosomal acidification decreases DENV infection, neural impairment, and mortality in suckling mice

**Figure 6:**
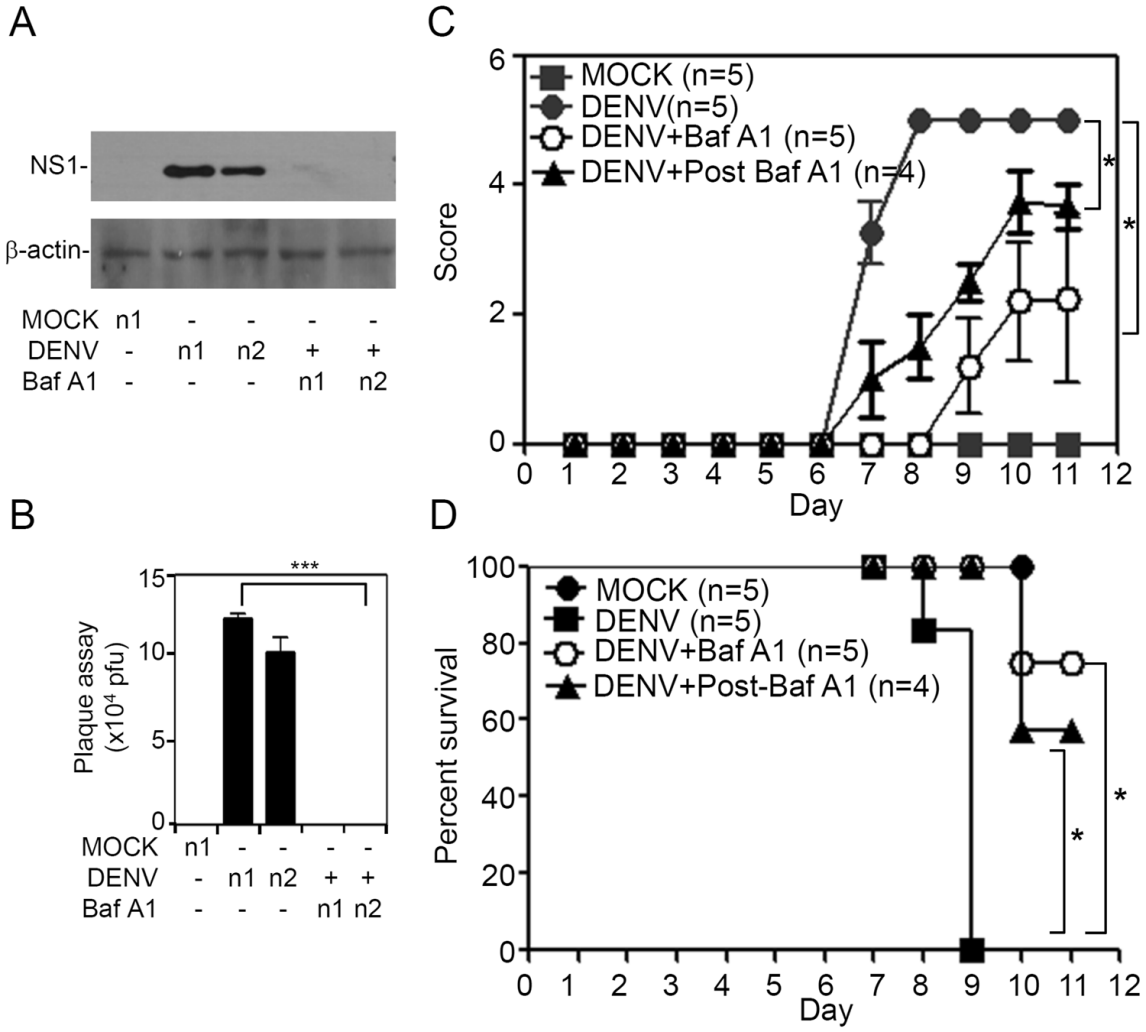
Inhibiting endosomal acidification increases the survival rate in suckling mice during dengue virus (DENV) infection. Seven-day-old ICR suckling mice were inoculated with DENV2 by concurrent intracranial and intraperitoneal injections with or without bafilomycin A1 (BafA1; 1 mg/kg) co-treatment or post-treatment. (**A**) Western blot analysis of viral NS1 or NS4B protein expression and (**B**) plaque assays of DENV replication in the brain of ICR suckling mice at 7 days post-infection. Values are presented as the mean ± SD (*n* = 4~7). ***p < 0.001. Additionally, time-kinetic changes in clinical scores (**C**) and survival rates (**D**) were measured (*n* = 4 or 5). *p < 0.05.

Our previous animal model of DENV infection used immunocompetent mice of the ICR strain to induce viral replication in the brain causing acute encephalitis^16^. BafA1 was administered at days 0 and 1 post-infection. We exploited a series of methods, including a Western blot analysis of the NS1 and NS4B viral proteins (Fig. 6A) and plaque assays for detecting virus replication (Fig. 6B). According to our results, DENV caused significant infection and replication in mouse brains at 7 days post-infection, as BafA1 inhibited viral protein expression and replication. Mice with neurological changes were then evaluated as previously described^16^. We monitored time-dependent changes in clinical scores, which were graded according to the severity of illness as follows: 0 for healthy; 1 for minor illness, including weight loss, reduced mobility, and a hunchback body orientation; 2 for limbic seizures; 3 for moving with difficulty and anterior limb or posterior limb weakness; 4 for paralysis; and 5 for death. A significant increase in clinical scores (Fig. 6C) had occurred in DENV-infected mice compared to mock-infected mice by 7 days post-infection. The survival rate of DENV-infected mice had decreased by days 8 or 9 post-infection, and all of the mice had died by days 9 or 10 post-infection (Fig. 6D). Either co- or post-treatment with BafA1 significantly reduced DENV-induced disease progression and mortality. The data indicated that inhibiting endosomal acidification abolished encephalitic DENV infection in our model, leading to neural impairment following viral replication.

## Discussion

Neurological complications of DENV infection are now classified as some of the hallmarks of severe dengue. In addition to our *in vitro* study showing DENV-induced MOI- and time-dependent neurotoxicity, our previous study indicated that DENV caused *in vivo* infection in neuronal cells among hippocampal lesions and induced neuronal cell apoptosis^16^. We further showed that a clathrin-regulated endocytic pathway controls viral entry at an early step of infection in neuronal cells. Following endocytosis, lysosomal acidification is essential for DENV genome uncoating and replication in infected neuronal cells. Regarding no further studies showing the blockade of endocytic pathways for anti-DENV actions in mice, this study demonstrated that inhibiting the endocytic pathways of DENV infection decreased viral replication and attenuated DENV-induced neurotoxicity as well as acute viral encephalitis.

In general, neurological complications associated with dengue diseases are unusual. Treatment with PCZ, a D2R antagonist that has been approved for treating nausea, vomiting, and headaches in humans, confers anti-dengue effects *in vitro* and *in vivo* ^19^. As to viral entry, PCZ can also act as an inhibitor of clathrin-mediated endocytosis. The roles of PCZ in targeting D2R and clathrin during DENV infection need further investigation. In addition to PCZ, the anti-psychotics, CPZ and Pitstop 2, were used in this study to confirm the essential role of clathrin-mediated endocytosis in DENV entry. CPZ is also an antagonist of D2R^29^. We showed that D2R was expressed in DENV-infected neuronal cells and mediated DENV entry as a viral receptor. All of these studies indicated that neuronal cells can be targets of DENV infection through potential viral receptor D2R-mediated and clathrin-regulated viral entry. Serotype 2 of DENV was used in our work as well as in a previous study^19^; however, further studies have to dissect the different routes of clathrin-dependent and -independent endocytosis by other, different serotypes of DENV^4^. Importantly, according to *in vivo* and *in vitro* immunostaining, D2R was expressed in hippocampal neurons, consistent with a previous study^17^. The pathogenic effects of DENV-infected hippocampal neurons remain unclear in dengue encephalitis. The affinity of viral infection and neurotoxicity, especially in the hippocampal regions, is of interest for further studies.

In addition to developing vaccines and viral inhibitors that precisely target viral proteins, which are essential for viral binding/entry, replication, and assembly/release, identifying specific virus-host interactions, such as viral receptors, endocytic pathways, and viral assembly, could be useful for anti-infective therapies^3, 5, 6^. Although the search for antivirals to combat DENV infection is critical, there are no currently accepted antiviral drugs for treating dengue patients. Previous studies showed that the administration of chloroquine, a lysosomotropic agent, exerts a modest antiviral effect by interfering with endosomal fusion and furin-dependent virus maturation *in vitro* ^30^ and *in vivo* ^31^. It was speculated that chloroquine and its analogue, hydroxychloroquine, could be used to treat low pH-dependent viruses, such as dengue, chikungunya, influenza, and Ebola, at the initial phase of infection^32, 33^. However, a randomized controlled trial of chloroquine failed to inhibit viremia, antigenemia, and cytokine or T cell responses in dengue patients^34^. Further studies may be needed to evaluate its therapeutic efficacy and treatment route.

By small interfering RNA screening, several human membrane trafficking genes that mediate the infectious entry of DENV were identified^35, 36^. Importantly, knockdown of V-ATPase reduced DENV infection and replication in both arthropod cells^36^ and human cells^35^. Studies in arthropod vectors or cells showed blockade of the endocytic pathway of DENV infection by BafA1, an inhibitor of V-ATPase, thereby reducing viral replication and transmission^36–38^. According to our study, targeting V-ATPase-based endosomal acidification using BafA1 and ConA, which bind to the proteolipid ring of the V_0_ domain of V-ATPase^28^, resulted in a notable antiviral response against DENV replication *in vitro* and *in vivo*. Consistent with a previous study as demonstrated *in vitro* ^35^, our study further showed both *in vitro* and *in vivo* antiviral effects of BafA1 treatment. These findings provide evidence to strengthen the preclinical importance of BafA1-based anti-dengue therapy.

Viral infections require an acidic pH for infectivity, generally during the process of viral genome uncoating following endocytosis. BafA1 can block V-ATPase-based endosomal acidification, and the blockade of endocytic pathways by BafA1 treatment can be demonstrated by detecting viral genome uncoating, protein expression, replication, and virus release^39^. In our study, treatment with BafA1 significantly decreased DENV RNA replication, protein expression, and virus release *in vitro* and *in vivo*. These results confirmed the importance of endocytic pathways for DENV infection. However, the antiviral efficiency of BafA1 could also include targeting the autophagic process^40^, as autophagy-regulated energy production is required during DENV replication^41^. However, in this study, as demonstrated using a stable luciferase reporter DENV, BafA1 treatment did not affect viral translation, which suggests that its antiviral action presumably occurs by means other than by directly affecting viral replication-associated autophagy. Furthermore, V-ATPases are not only found within membranes of endosomes, lysosomes, and secretory vesicles, but are also found in plasma membranes^28^. It was speculated that BafA1 treatment may also affect upstream and downstream aspects of the endocytic pathways of DENV infection, such as viral entry or the secretion of infectious virions.

Neurotoxicity can be caused by DENV infection in brains of experimental mice^14, 16, 24^, and DENV infection has caused fatalities in dengue patients^42^. Several *in vitro* studies showed the ability of DENV infection to trigger neuronal cell death^20–23^. We confirmed the cytotoxic effects caused by DENV infection in neuronal cells *in vivo* and *in vitro*. After either a long time post-infection or a higher MOI, DENV caused neuronal cell growth inhibition accompanied by cytotoxicity. In response to viral replication, cellular stress, including endoplasmic reticular stress, oxidative stress, and cytotoxic factor release (such as tumor necrosis factor-α), may promote the apoptosis of neuronal cells. A direct effect of DENV infection on cytotoxicity in neuronal cells was speculated *in vitro*; however, a bystander effect such as CNS inflammation, which is probably caused by DENV infection of microglia and astrocytes, may also contribute to neurotoxicity *in vivo*.

In conclusion, targeting the endocytic pathways of DENV infection can reduce viral replication and cytotoxicity in neuronal cells *in vitro* and *in vivo*. These findings shed light on the development of antiviral therapeutics against DENV infection, particularly in the brain. In addition to DENV, targeting an Axl-mediated clathrin-based endocytic pathway conferred protection against the emerging flavivirus Zika infection in human glial cells^43^. Similar to flaviviruses, endosomal acid-dependent viral entry may also determine Zika membrane fusion during early infection and could be targeted for further antiviral therapy. For flaviviral encephalitis, in addition to a direct cytotoxic effect on neuronal cells, immunopathogenic issues, including innate and adaptive immune responses in the CNS, are also involved in encephalitic development^44^. Questions have been raised by this study that require further examination, including the involvement and interaction of DENV-infected cells, post-infection effects of neuronal cells, and other host factors involved in DENV infection, neurotoxicity, and dengue encephalitis.

## Methods

### Ethics statement

Animal studies of this project were performed according to the *Animal Protection Act* of Taiwan, and all protocols according to guidelines established by the Ministry of Science and Technology, Taiwan were approved by the Laboratory Animal Care and Use Committee of National Cheng Kung University (IACUC #104062).

### Cells, virus strains, and reagents

Mouse Neuro-2a cells (ATCC, CCL131) were grown on plastic in RPMI medium 1640 (RPMI; Invitrogen Life Technologies, Rockville, MD) supplemented with 10% heat-inactivated fetal bovine serum (FBS; Invitrogen Life Technologies). Baby hamster kidney (BHK)-21 cells (ATCC, CCL10) and *Aedes albopictus* C6/36 cells (ATCC, CRL1660) were cultured in Dulbecco’s modified Eagle’s medium (DMEM; Invitrogen Life Technologies). DENV2 PL046, a Taiwanese human isolate obtained from the Centers for Disease Control in Taiwan, was propagated in C6/36 cells. Viral titers were determined by plaque assays using the BHK-21 cell line. Reagents and antibodies used in these studies were as follows: chlorpromazine (CPZ) and bafilomycin A1 (BafA1) (Cayman Chemical, Ann Arbor, MI); 4,6-diamidino-2-phenylindole (DAPI), acridine orange, dimethyl sulfoxide (DMSO), concanamycin A (ConA), MCP, and a mouse monoclonal antibody (mAb) specific for β-actin (Sigma-Aldrich, St. Louis, MO); antibodies against dsRNA (Scicons, city?, Hungary); antibodies against NeuN, DENV NS1, and NS4B (GeneTex, San Antonio, TX); Pitstop 2 and rabbit anti-mouse immunoglobulin G (IgG) conjugated with horseradish peroxidase (HRP; Abcam, Cambridge, MA); and Alexa Fluor 488- and Alexa Fluor 594-conjugated goat anti-mouse and goat anti-rabbit [?]antibodies (Invitrogen, Carlsbad, CA).

### Isolation of neuronal cells

Hippocampal neurons were dissected from E16.5 C57BL/6 mouse embryos (Jackson Laboratory, Bar Harbor, MA) and suspended in 0.02% trypsin-EDTA (Invitrogen) at 37 °C for 10 min according to a previous study^45^.

### DENV infection *in vitro* and *in vivo*

The *in vitro* and *in vivo* infectious procedures were carried out according to our previous studies^16, 46^. Seven-day-old ICR strain suckling mice were inoculated intracerebrally with 2.5 × 10^5^ plaque-forming units (PFU) and intraperitoneally with 7.5 × 10^5^ PFU of DENV2 (PL046), which was combined with or without BafA1 (1 mg/kg) treatment.

### Immunostaining

Procedures were carried out according to our previous studies^16^. For actin staining, Alexa Fluor 594 phalloidin (Thermo Fisher Scientific, Pittsburgh, PA) was used. Cells were visualized under a fluorescent microscope (BX51; Olympus, Tokyo, Japan) or a laser-scanning confocal microscope (SPII; Leica Mikrosysteme Vertrieb, Bensheim, Germany). Cells were analyzed using flow cytometry (FACSCalibur; BD Biosciences, where?).

### TdT-mediated dUTP nick end labeling (TUNEL) assay

Apoptotic cells were assessed by TUNEL staining using an ApoAlert DNA fragmentation assay kit (Clontech, Mountain View, CA) according to the manufacturer’s instructions.

### Fluorescent DENV

Fluorescent DENV was prepared by labeling with Alexa Fluor 594 succinimidyl ester (AF594SE, Molecular Probes, Invitrogen) according to a method described in a previous study^47^. Labeled viruses were purified using Amicon Ultra-15 PLTK Ultracel-PL Membrane (30 kDa) centrifugal filter units (Millipore, where?) to remove excess dye.

### Cell viability and cytotoxicity

Cell viability and cytotoxicity were respectively assessed using a colorimetric Cell Counting Kit-8 (Dojindo Molecular Technologies, Kumamoto, Japan) and Cytotoxicity Detection kit assays (Roche Diagnostics, Lewes, UK), according to the manufacturer’s instructions.

### Western blotting

The general protocol for Western blotting was performed according to previous studies^16, 46^.

### Reverse-transcription (RT)-polymerase chain reaction (PCR) and quantitative (q)PCR

Total RNA was extracted using the TRIZol (Invitrogen) RNA extraction reagent. Complementary (c)DNA was synthesized with an RT reaction using a PrimeScript^TM^ RT reagent kit (Takara, Tokyo, Japan). The qPCR was conducted using KAPA SYBR FAST qPCR Master Mix (Life Technologies and Kapa Biosystems, Woburn, MA). The PCR was performed using a StepOnePlus^TM^ real-time PCR system (Applied Biosystems, Foster City, CA) with the following pair of specific primers: primer sequences for *NS1* (forward): 5′-ATGGATCCGATAGTGGTTGCGTTGTGA-3′ and *NS1* (reverse): 5′-ATCTCGAGGGCTGTGACCAAGGAGTT-3′.

### Plaque assay

Detecting viral replication was performed with a plaque assay according to previous studies^16, 46^.

### Reporter assay

BHK-21 cells harboring the luciferase-expressing DENV replicon (BHK-D2-Fluc-SGR-Neo-1) were constructed and maintained according to a previous study^48^.

### Statistical analysis

Data are presented as the mean ± standard deviation (SD). Data were analyzed by an unpaired Student’s *t*-test or by one-way analysis of variance (ANOVA) with Tukey’s multiple-comparison test. Statistical significance was defined as *p* < 0.05.

## Electronic supplementary material

Supplementary information
