# Supplementary material for: Blockade of dengue virus infection and viral cytotoxicity in neuronal cells in vitro and in vivo by targeting endocytic pathways
Source: Sci Rep. 2017 Jul 31;7:6910. doi: 10.1038/s41598-017-07023-z (PMC5537343; doi:10.1038/s41598-017-07023-z)

Blockade of dengue virus infection and viral cytotoxicity in neuronal cells *in vitro* and *in vivo* by targeting endocytic pathways

Min-Ru Ho, Tsung-Ting Tsai, Chia-Ling Chen, Ming-Kai Jhan, Cheng-Chieh Tsai, Yi-Chao Lee, Chun-Han Chen, and Chiou-Feng Lin

Supplemental information

Supplemental Figure 1 DENV2 causes D2R-mediated infection. Flow cytometry and immunofluorescent analysis showed Neuro-2a (**A**) and primary cultured hippocampal neuronal cells (**B**) expressing D2R or D4R. (**C**) Primary cultured hippocampal neuronal cells were inoculated with Alexa-594 labeled (*red*) DENV2 (MOI=1) for 2 h. The fluorescent image shows cells carrying fluorescent DENV2. (**D**) Plaque assays showed the level of viral replication. Neuro-2a cells were infected with DENV2 (MOI=1) with or without D2R inhibitor MCP (10 μM) pre-treatment. (**E**) The fluorescent image shows cells carrying Alexa-594 labeled (*red*) DENV2 2 h post-infection. (**F**) Plaque assays show the level of viral replication. For all images and histograms, representative data were selectively obtained from three individual experiments. DAPI staining indicates the nuclei (*blue*). For flow cytometry analysis, the percentage of positive cells is shown. All quantitative data are shown as the means ± SD from three independent experiments. *$p < 0.05$, **$p < 0.01$, and ***$p < 0.001$. ns, not significant.

Ho et al Supplemental Figure 1

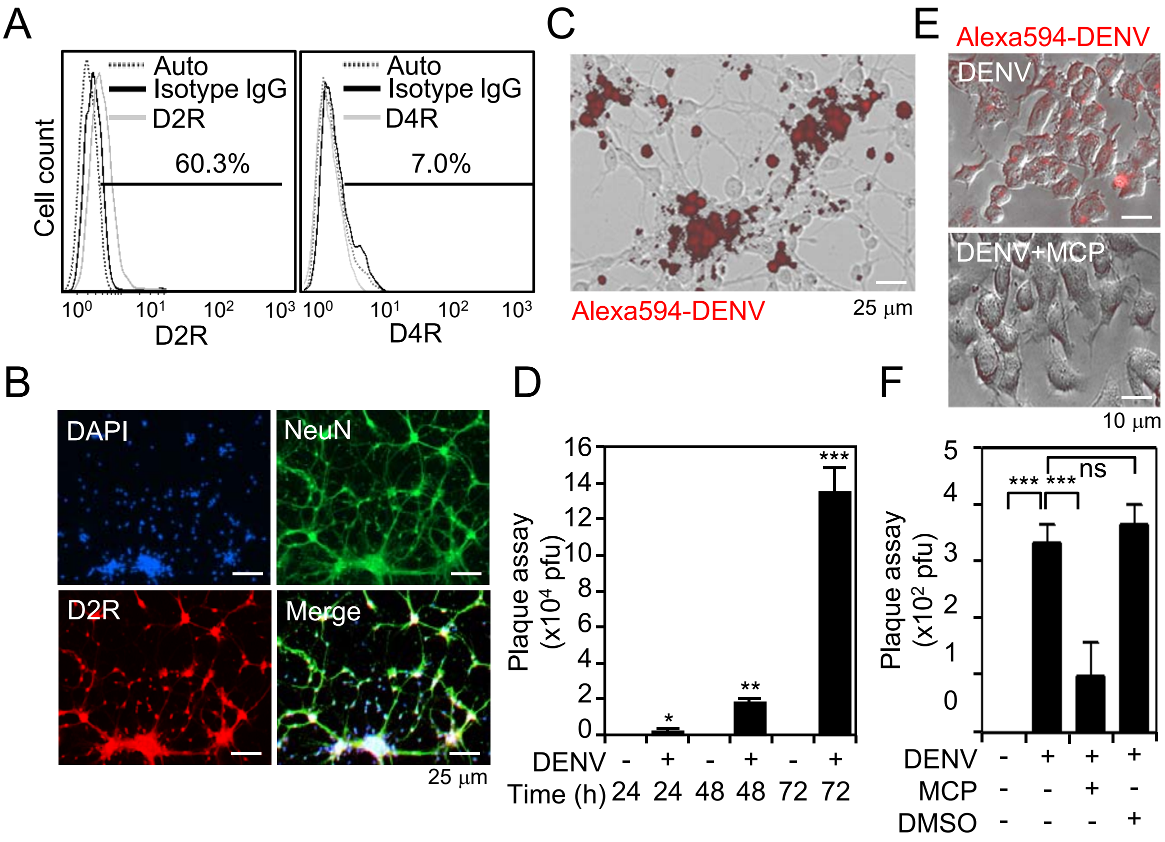

Supplement: Supplementary file 1 — Supplementary information [file 41598_2017_7023_MOESM1_ESM.pdf]
